# Chemically Engineered Sulfates of Lasiodiplodan: Antioxidant, Antimicrobial, and Antirespiratory Syncytial Virus (RSV) Activities

**DOI:** 10.1021/acsomega.5c11984

**Published:** 2026-03-23

**Authors:** Alaor Martins da Silva, André Luiz Dyna, Tamires Pereira Rosa, Gabrielle Cristina Calegari, Alexandre Orsato, Aneli M. Barbosa-Dekker, Robert F. H. Dekker, Ligia Carla Faccin-Galhardi, Mário Antônio Alves da Cunha

**Affiliations:** † Postgraduate Program in Chemical and Biochemical Process Technology, Federal University of Technology - Paraná, Pato Branco 85503-390, Brazil; ‡ Department of Microbiology, State University of Londrina, Londrina, Paraná 86057-970, Brazil; § Department of Chemistry, State University of Londrina, Londrina, Paraná 86057-970, Brazil; ∥ Beta-Glucan Produtos Farmoquímicos, Federal University of Technology - Paraná, Londrina, Paraná 86036-700, Brazil

## Abstract

β-Glucans are carbohydrate macromolecules with significant clinical and biotechnological applications, whose biological properties are enhanced through chemical modifications, such as sulfation. This study aimed to chemically engineer sulfated derivatives of the (1→6)-β-d-glucan lasiodiplodan and to evaluate how different degrees of sulfation influence its antioxidant, antimicrobial, and antiviral activities. The chemically engineered sulfated derivatives LAS-S1 (DS 0.11) and LAS-S2 (DS 0.51) were synthesized using the sulfating agent chlorosulfonic acid, with different solvents: *N*,*N*-dimethylformamide (solvent/stabilizer) and pyridine (proton-neutralizing agent). Sulfation improved the solubility and enhanced the biological activities of both derivatives. LAS-S2 demonstrated better antimicrobial and antiviral activity than LAS-S1. Sulfation was shown to contribute to ·OH and H_2_O_2_ scavenging activity, with a dose-dependent effect related to both the concentration of the sulfated compound and its degree of sulfation. The fungistatic and bacteriostatic effects were demonstrated against *Escherichia coli*, *Listeria monocytogenes*, *Salmonella enterica* Typhimurium, *Candida albicans*, and *Candida tropicalis*. LAS-S2 exhibited potent inhibition of respiratory syncytial virus (RSV) in vitro, with a high selectivity index (>724.63), and interfered with multiple stages of viral replication. These findings highlight sulfation as an effective strategy to enhance the biological functions of lasiodiplodan. LAS-S2 emerges as a safe (noncytotoxic) and promising candidate for biotechnological and pharmaceutical applications, including novel antimicrobial and antiviral treatments. Future studies should focus on elucidating structure–activity relationships, optimizing sulfation patterns, and evaluating the in vivo efficacy and pharmacological properties of sulfated lasiodiplodan derivatives.

## Graphical abstract

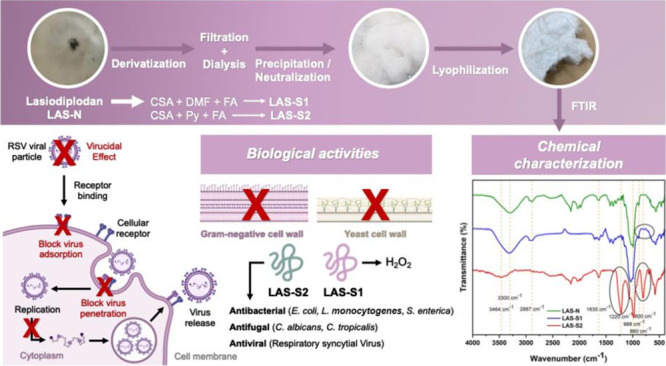

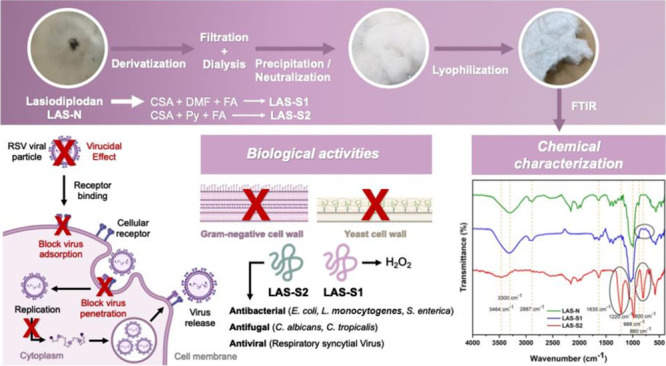

## 1 Introduction

Respiratory syncytial virus (RSV) is an enveloped virus that contains a coat bearing glycoproteins, through which the virus binds to host cell receptors, initiating pathologic infections in both the upper and lower respiratory tracts. These infections typically cause mild symptoms that usually resolve within 2 weeks. RSV possesses a negative-sense, single-stranded RNA genome with 10 genes that encode 11 viral proteins and are classified into two antigenically distinct subtypes.^1^ RSV represents a global endemic threat, affecting approximately 64 million cases yearly, including children and adults.^2^ RSV is one of the main viruses widespread in early childhood and is associated with severe human respiratory infections and high rates of hospitalization and mortality in all age groups worldwide. In young children (<5 years old), the infection is associated with severe and potentially fatal diseases (bronchiolitis and viral pneumonia), while the elderly (>60 years old) and immunocompromised individuals are also potentially at risk from the virus.^1^

Despite the availability of recent FDA/USA-approved vaccines and monoclonal antibodies to treat higher-risk patients, the high prevalence and adverse effects of available treatments encourage the search for new anti-RSV therapeutic alternatives.^3–5^ In addition to viral infections, bacterial and fungal infections remain a major threat to public health as they are becoming increasingly common and resistant to available antimicrobial treatments.^6^ In 2019, antimicrobial resistance was responsible for 1.27 million deaths, a figure projected to rise to 10 million annually by 2050, surpassing cancer-related deaths.^7^ Therefore, research to discover new antiviral and antimicrobial compounds that are not prone to adverse side effects is considered to be vitally important.

Carbohydrate biopolymers such as polysaccharides, and especially exopolysaccharides (EPS) produced as secondary metabolites by microorganisms, have demonstrated several interesting biological properties, including antitumor, anti-inflammatory, antioxidant, antibacterial, and antiviral activities^8^ Several studies^9^ have described the antiviral activity of natural or semisynthetic sulfated polysaccharides and their efficacy in altering viral replication or cellular responses to infection. In this context, the EPS, lasiodiplodan, is an uncommon linear-linked (1→6)-β-d-glucan from *Lasiodiplodia theobromae* isolate MMPI^10^ that belongs to the family of fungi (ascomycetes) representative of the order Botryosphaeriales (Botryosphaeriaceae). The chemical structure of native LAS (LAS-N) allows modifications that alter its physicochemical properties (including polarity and solubility) and confer new biological properties upon sulfation.

The biological activities of lasiodiplodan (LAS) are well-documented, and among them are antiproliferative effects in MCF-7 human breast carcinoma cells,^11^ in which apoptosis was mediated by AMP-activated protein kinase (AMPK) and Forkhead transcription factor, FOXO3a;^12^ protection against doxorubicin-induced DNA damage;^13^ prevention of neurotoxicity signals in rat cerebral cortex induced by D-penicillamine and behavioral signs of convulsive episodes related to the GABAergic system;^14^ subchronic treatments of Swiss mice with lasiodiplodan over 28 days lowered blood glucose levels in the male group only and reduced blood cholesterol levels in both genders, but such treatments did not lead to toxicity in either gender;^15^ and finally, induction of collagen synthesis during wound healing when lasiodiplodan was used as an active ingredient in a hydrogel.^16^ In addition, sulfation of lasiodiplodan (LAS-S) demonstrated a higher antimicrobial potential against Gram-positive and Gram-negative bacteria and some yeasts compared to native LAS.^17^ Sulfation further expands its spectrum of action, conferring anticoagulation,^18^ antimicrobial (*Candida albicans* and *Salmonella enterica*),^19^ and antiviral (Herpes simplex HSV-1) activities.^20^

Seeking to generate innovative knowledge aimed at the development of new antiviral drugs, this study reports on chemically engineering LAS by sulfation using chlorosulfonic acid (CSA) by two protocols employing the properties of two different solvents, N,N-dimethylformamide (DMF) and pyridine (Py): i.e., (1) CSA-DMF and (2) CSA-Py. Two new sulfated polysaccharides (LAS-S1 and LAS-S2) were obtained from LAS-N and chemically characterized, and their antioxidant, antimicrobial (*S. aureus*, *L. monocytogenes*, *E. coli*, *S. typhimurium*, *C. albicans*, and *C. tropicalis*), and antiviral (respiratory syncytial virus, RSV) activities were evaluated.

## 2 Material and Methods

Analytical-grade solvents and reagents were obtained from Sigma-Aldrich (St. Louis, MO, USA). Glucose, the mineral salts used for fermentation media preparation, and Sabouraud agar culture medium were purchased from Synth Company (Brazil).

### 2.1 Production of Lasiodiplodan

A portion of the stock culture of *L. theobromae* MMPI mycelium was transferred to Petri dishes containing Sabouraud-chloramphenicol agar to prepare the inoculum, as previously described by Cunha et al.^11^ Submerged cultivation was carried out in 250 mL Erlenmeyer flasks containing 100 mL of Vogel minimal salts medium (VMSM) supplemented with 20 g/L glucose and 10 mL of inoculum. After incubation (28 °C) under orbital shaker conditions (150 rpm) for 72 h, the mycelium was separated by filtration, and the filtrate containing the EPS (lasiodiplodan) was precipitated out of solution with three volumes of ethanol (95%, v/v) at 5 °C overnight. The precipitate was collected and dissolved in distilled water by heating at 60 °C, followed by intense dialysis for 5 days using a cellulose membrane with a molecular weight cutoff (MWCO) of 6–8 kDa (Spectrum Laboratories, USA), with three daily exchanges of distilled water. After dialysis, the material was lyophilized to obtain LAS-N, which was stored in airtight vials under refrigeration for subsequent sulfation.

### 2.2 Chemical Derivatization of Lasiodiplodan by Sulfation

LAS-N was sulfated using two protocols, 1 ^21^ and 2.^22^ Protocol 1 via SO_3_-DMF: 16.7 mL of chlorosulfonic acid (CSA) was slowly added to 100 mL of *N*,*N*-dimethylformamide (DMF) in an ice bath, forming the SO_3_-DMF complex, where sulfur trioxide (SO_3_) acts as the donor of the sulfate group (O–SO_3_H). In parallel, 500 mg of DW of lyophilized LAS-N was dissolved in 20 mL of formamide (FA) and stirred at 60 °C for 30 min. The SO_3_-DMF reagent (6.2 mL) produced above was then added to the LAS-N solution, and the reaction was maintained at 60 °C for 4 h. After this period, the mixture was cooled in an ice bath, and the pH was adjusted to 8.0 with 4 mol/L NaOH. The resultant sulfated derivative (LAS-S1) was precipitated with 95% ethanol (volume 3:1) at 5 °C for 24 h. The precipitate was collected, dissolved in 100 mL of distilled water, and dialyzed using a 6–8 kDa membrane for 96 h (48 h in running water and 48 h in distilled water). The resulting solution was then lyophilized, yielding the LAS-S1 compound.

Protocol 2 via SO_3_–Py: 3.0 mL of CSA was slowly added to 24.0 mL of pyridine (Py) while being stirred in an ice bath, forming the chlorosulfonic-pyridine salt (SO3-Py) as an intermediate. In parallel, 500 mg of lyophilized LAS-N was dissolved in 50 mL of FA while stirring at 25 °C for 30 min. Next, 20 mL of the SO_3_–Py compound was added dropwise to the LAS-N solution, and the reaction mixture was maintained at 80 °C for 3 h. Neutralization was performed with 15% (w/v) NaOH to a pH of 7.0, followed by precipitation with ethanol 95% (volume 3:1) at 5 °C for 24 h. The LAS-S2 product was recovered, dialyzed under the same conditions as in Protocol 1, and then lyophilized. Both sulfation protocols are summarized in Figure 1.

**Figure 1:**
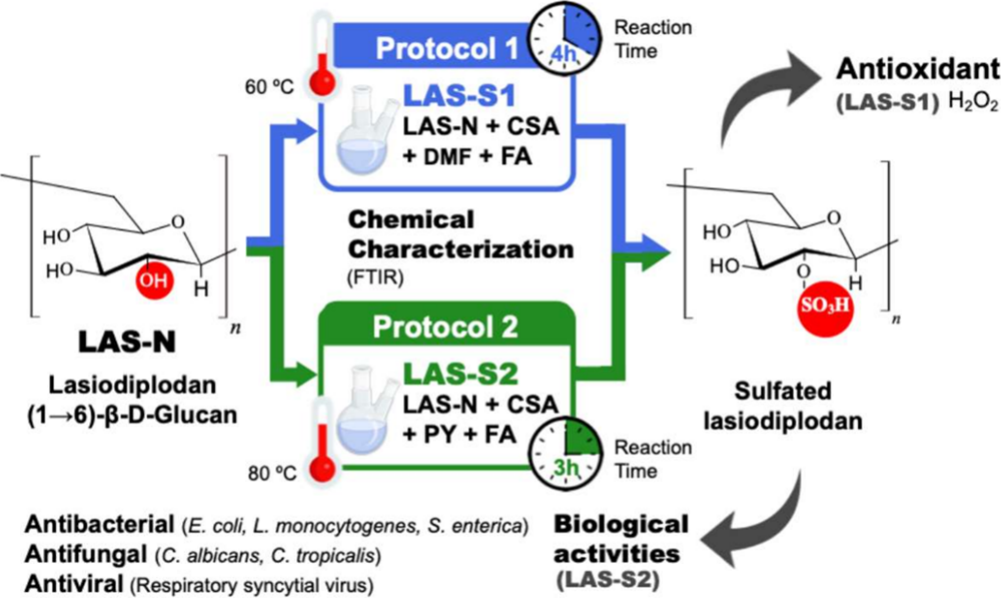
Schematic flowchart of chemical derivatization by sulfation of native lasiodiplodan by Protocol 1 (CSA-DMF-FA) and Protocol 2 (CSA-Py-FA).

### 2.3 Determination of the Degree of Sulfation and Solubility of Sulfated Lasiodiplodan

The degree of sulfation (DS) was determined by correlating the sulfur and carbon contents of the sample, as described by Moura Neto et al.^23^ The analyses were conducted using a CHNS/O 2400 Series II Elemental Analyzer (PerkinElmer, USA). The degree of sulfation refers to the average number of groups of O–SO_3_H attached to each monosaccharide unit of the polysaccharide.

For the evaluation of water solubility, the protocol described by Wang et al.^24^ was employed with adaptations. Samples of 10 mg of LAS-1 and LAS-2 were suspended in 10 mL of distilled water and agitated for 24 h at 25 °C. The samples were then centrifuged at 1500*g* for 10 min. The collected supernatant was used to quantify the total sugar content by the phenol-sulfuric acid method,^25^ which is directly related to the amount of soluble mass. Water solubility was expressed as the percentage of the soluble lasiodiplodan mass. Solubility in 20% dimethyl sulfoxide (DMSO) was evaluated by using the same method, substituting water with 20% DMSO, prior to the biological activity assays.

### 2.4 FTIR Spectroscopy

The lasiodiplodan samples (both native and sulfated) were characterized by Fourier-transform infrared spectroscopy (FTIR). FTIR spectra were obtained using a Frontier spectrometer (PerkinElmer, USA) in the range of 400–4000^–1^ at a resolution of 2 cm^–1^. Each spectrum was acquired by averaging 16 scans using the attenuated total reflection (ATR) technique.

### 2.5 Determination of Antioxidant Properties

The antioxidant potential of the LAS compounds was evaluated based on their ability to scavenge the hydroxyl radical (·OH) and hydrogen peroxide (H_2_O_2_), as reported by Liu et al.,^26^ with minor modifications.

Scavenging of the hydroxyl radical (·OH): the assay was performed using a reaction mixture containing 0.5 mL of FeSO_4_ (1.5 mmol/L), 0.35 mL of H_2_O_2_ (6 mmol/L), 0.15 mL of sodium salicylate (20 mmol/L), and 1 mL of the LAS compounds at various concentrations (25, 15, and 10 mg/mL). After incubation for 1 h at 37 °C, absorbance was measured at 562 nm using a UV–vis spectrophotometer (KASVI, Brazil).

Scavenging of hydrogen peroxide (H_2_O_2_): the assay was performed with a reaction mixture containing 1 mL of freshly prepared H_2_O_2_ (0.1 mmol/L), 1 mL of LAS sample suspensions (150 mg/L), 0.1 mL of ammonium molybdate (3% w/v), 10 mL of H_2_SO_4_ (2 mol/L), and 7 mL of KI (1.8 mol/L). The mixture was titrated with Na_2_S_2_O_3_ (5 mmol/L) until the yellow color disappeared. Ascorbic acid (AA) was used as a positive control (antioxidant standard) for both assays.

### 2.6 Antibacterial and Antifungal Activities

The antibacterial and antifungal activities were evaluated using the microdilution method to determine the minimum inhibitory concentration (MIC) against bacteria (*Escherichia coli* ATCC 25922, *Listeria monocytogenes* ATCC 19111, *Staphylococcus aureus* ATCC 25923, and *Salmonella enterica* Typhimurium ATCC 0028), and yeasts (*Candida albicans* ATCC 10231 and *Candida tropicalis* 13803). For the antimicrobial assays, 96-well plates were prepared by adding 100 μL of LAS-N, LAS-S1, or LAS-S2 solutions at decreasing concentrations (0.25, 0.20, 0.15, 0.10, and 0.05 mg/mL), 100 μL of Mueller–Hinton broth (for bacteria) or Sabouraud broth with chloramphenicol (for yeasts), and 5 μL of a standardized microbial suspension (McFarland scale of 0.5; 1.5 × 10^8^ CFU/mL). Plates were incubated in a bacteriological incubator (Novatecnica, Brazil) at 37 °C for bacteria, or 28 °C for yeasts. After 24 h of incubation at the respective temperatures, 20 μL of a 0.01% (w/v) resazurin dye solution was added to each well, and the mixtures were incubated for an additional 2 h to assess microbial cell viability.

Blue-stained wells indicated no reduction of resazurin and, consequently, no metabolic activity of the microbial cells (evidence of effective antimicrobial action). Pink-stained wells demonstrated that metabolically active cells (viable bacteria) reduced the amount of dye to resorufin. Broths showing positive inhibition were streaked onto Brain Heart Infusion agar for bacteria or Sabouraud agar supplemented with chloramphenicol for yeasts to verify microbiostatic (inhibition of microbial growth) or microbicidal (death of the microbial cell) activity after incubation at 37 °C (bacteria) or 28 °C (yeasts) for 24 h. Tetracycline and fluconazole were used as positive controls for bacteria and yeasts, respectively, while sterile 0.1% peptone water served as the negative control.

### 2.7 Antiviral Activity Assessment

Antiviral activities of native lasiodiplodan (LAS-N) and the derivatized lasiodiplodans (LAS-S1 and LAS-S2) were tested against respiratory syncytial virus (RSV, strain long (hRSV A), propagated in human laryngeal carcinoma cells (HEp-2; ATCC CCL-23, USA). The polysaccharides were solubilized in Dulbecco’s Modified Eagle Medium (DMEM, Merck, Germany) and stored at −20 °C. HEp-2 cells were cultured (1 × 10^6^ cells) in DMEM supplemented with 2 mM glutamine (Merck), 10% fetal bovine serum (Bionutriente, Brazil), 2.5 μg/mL amphotericin B (Novafarma, Brazil), 100 μg/mL streptomycin (Merck), and 100 IU/mL penicillin (Novafarma) and maintained at 37 °C under 5% CO_2_ (Sanyo, Japan). Viral stock was stored at −80 °C in the presence of 25% (w/v) sucrose solution. The RSV inhibitor, ribavirin (Jinan Mingxin, China), a purine nucleoside analogue, was used as a positive control and was solubilized in serum-free DMEM (4 mg/mL, Merck) and stored at −20 °C.

#### 2.7.1 Cytotoxicity Assay

The cytotoxicity of the LAS compounds was evaluated using the MTT (3-(4,5-dimethylthiazolyl-2)- 2,5-diphenyltetrazolium bromide) assay.^27^ Hep-2 cells (3 × 10^4^ cells/well) cultured in 96-well microplates were treated with the LAS samples at concentrations ranging from 62.5 to 1000 μg/mL and incubated for 72 h at 37 °C with 5% CO_2_. Cells containing only DMEM-SF were maintained as a negative control. Following incubation, 10 μL of MTT solution (0.5 mg/mL, Life Technologies, USA) was added to each well. After 4 h, the medium was removed, and 900 μL of solubilization solution (acidified isopropanol containing 0.01 mol/L HCl and 0.36% (v/v) Triton-X) was added to dissolve the formazan crystals formed during the MTT assay. Absorbance was measured at 570 and 690 nm using a microplate reader (Bio-Tek Instruments, USA), and the results were expressed as percentages of cell viability. The half-maximal cytotoxic concentration (CC_50_) was calculated using regression analysis.

#### 2.7.2 Antiviral Screening Assay

The antiviral screening was performed using the TCID_50_ (tissue culture infectious dose at 50%) and MTT reduction assays against RSV, as described by previous studies,^28,29^ with modifications. For antiviral screening, HEp-2 cells (3 × 10^4^ cells/well) were cultivated in 96-well plates, and the compounds LAS-N, LAS-S1, and LAS-S2 (12.5–200 μg/mL) were added simultaneously to the cells along with RSV (multiplicity of infection, MOI 0.1). Cells maintained with DMEM-SF and cells with DMEM-SF containing the viral inoculum were used as cell controls (CC) and virus controls (VC), respectively. The plates were incubated at 37 °C with 5% CO_2_ for 72 h. The percentage of viral inhibition (%VI) was calculated relative to CC and VC. The concentration that inhibits 50% of viral infectivity (IC_50_) was determined by linear regression, and the selectivity index (SI) was determined by the ratio between CC_50_/IC_50._ ^30^

#### 2.7.3 Mechanism of Action of Antiviral Activity

The sulfated polysaccharide with the best SI value was further investigated for its antiviral mode of action. The lasiodiplodan compounds (25–200 μg/mL) were added to cells and/or RSV (MOI 0.10) under different protocols. Viral controls (VC) and cell controls (CC) were included in all assays to ensure the validity of the results. The plates were incubated for 72 h, followed by the MTT assay as described previously. Protocols tested: (i) time of addition: the cells were exposed to the LAS compounds for 1 and 3 h before (pretreatment: −1 and −3 h), or for the same periods after (post-treatment: +1 and +3 h) viral inoculation; (ii) virucidal assays: the LAS compounds were incubated directly with the virus for 1 h, followed by dilution of the mixture 100-fold in DMEM and inoculation onto the cells; (iii) adsorption inhibition: the cells were preincubated at 4 °C for 30 min with the LAS compounds added simultaneously with the virus. Further incubation was performed at 4 °C for 90 min, followed by washing to remove unbound viral particles; and (iv) penetration inhibition: after preincubation at 4 °C for 30 min, the cells were inoculated with RSV and further incubated at 4 °C for 90 min. The cells were next washed to remove unabsorbed virus and treated with the LAS compounds for 10 min at 37 °C. The cells were then washed with Dulbecco’s phosphate-buffered saline (D-PBS) at pH 3.0 for 1 min, followed by buffer at pH 11.0 for 1 min, and incubated in DMEM for 72 h.^30^

### 2.8 Statistical Analysis

A two-way analysis of variance (ANOVA), followed by Dunnett’s multiple comparisons test, was applied to data sets with two independent variables. A *p*-value <0.05 was considered statistically significant. Results are presented as mean ± standard deviation from three independent experiments performed in triplicate. Statistical analyses were conducted using GraphPad Prism version 9.0. 0.191, 2021 (GraphPad Software, Inc., San Diego, CA, USA).

## 3 Results and Discussion

### 3.1 Sulfated Derivatives of LAS

Sulfation of lasiodiplodan by Protocol 1, where the SO_3_-DMF complex was used as the sulfating agent in formamide (FA) as the solvent, produced the LAS-S1 derivative with a low degree of substitution (DS: 0.11). By contrast, Protocol 2, where the chlorosulfonic-pyridine salt (SO_3_–Py) acted as the sulfating agent in formamide (FA) as the solvent, yielded the derivative LAS-S2 with an increased DS of 0.51 (Table 1).

**Table 1:** Degree of Substitution (DS) and Solubility of Lasiodiplodan Samples in Water and 20% DMSO at 25 °C^a^

		solubility (%)	
compounds	DS^b^	water	DMSO (20%)
LAS-N	0.00	6.15 ± 0.002	11.58 ± 0.002
LAS-S1	0.11	6.68 ± 0.002	7.73 ± 0.002
LAS-S2	0.51	17.38 ± 0.003	16.89 ± 0.002

a The results are expressed as means ± standard deviation. LAS-N: native lasiodiplodan. LAS-S: sulfated lasiodiplodans.

b Degree of substitution obtained.

Obtaining LAS derivatives with different DS values can be explained by a combination of chemical factors, including the reactivity of the sulfating agent, the nature of the intermediate formed, and reaction conditions (solvent, temperature, reaction time, final pH). In the SO_3_-DMF complex generated in Protocol 1, SO_3_ is stabilized by DMF. Therefore, a less reactive complex is formed, which limits the adequate availability of the sulfate group to react with the hydroxyl groups of LAS-N. In Protocol 2, pyridine acts as a Lewis base, facilitating the formation of a bond with chlorosulfonic acid and generating the pyridine-chlorosulfonic acid salt (SO_3_–Py), which is known to be a more efficient and reactive sulfating agent, releasing SO_3_ groups more easily and increasing the probability of substitutions in the macromolecule. These results align with the findings of Zhang et al.,^31^ using the CSA-Py method for sulfation of galactomannan from *Trigonella foenum-graecum* L., and achieved DS values of up to 0.49. Similarly, Chen et al.^32^ reported DS values ranging from 0.28 to 0.53 for two polysaccharides derived from *Cucurbita argyrosperma* (cushaw, an annual vine herb). Pires et al.,^33^ when comparing two methods for sulfation of chitosan using CSA-DMF, Method 1, which used only CSA and DMF, yielded a DS of 0.87, while Method 2, which included an additional DMF/formic acid step, produced a DS of 0.67.

The reaction temperature and time should also be mentioned. In our procedure, in Protocol 1, the reaction was conducted at 60 °C for 4 h, while in Protocol 2, 80 °C was used for over 3 h. Higher temperatures can increase the kinetic energy of the molecules and favor the accessibility and reactivity of the hydroxyl groups, resulting in higher DS values. Regarding the concentration and proportion of the sulfation reagents, it is noteworthy that in Protocol 1, only 6.2 mL of the sulfating agent SO_3_-DMF was added, whereas in Protocol 2, 20.0 mL of SO_3_-Py was used, thereby increasing the amount of sulfating agent available in the reaction mixture. Finally, pyridine should also be mentioned, which acts not only as an intermediate solvent but also as a stabilizing base, helping to neutralize protons released during sulfation. This prevents macromolecular degradation and maintains a greater number of free hydroxyl groups along the polysaccharide chain for sulfation.

d-Glucans, despite being polyhydroxylated macromolecules, exhibit low solubility due to the triple helical structure formed by interactions between the polyhydroxy groups in the β-d-glucan molecule, limiting their applications and reducing their in vivo physiological functions.^34^ Chemical modifications generally enhance solubility by altering intra- and intermolecular hydrogen bonding, thereby disrupting the triple helical conformation and promoting greater electrostatic repulsion. This allows the biopolymer chain to adopt a specific conformation in an aqueous solution. LAS-S1, with a low DS (0.11), showed only a slight increase in solubility in water and a decrease in 20% DMSO compared to native LAS-N. In contrast, LAS-S2 (DS 0.51) demonstrated higher solubility in both water and DMSO. These results suggest that sulfation with CSA-Py resulted in higher solubility, likely due to the distinct structural properties of the sulfating reagents, which promote better interaction with β-glucan and enhance the solubility of the polymer chain in the solvent.

#### 3.1.1 Fourier-Transform Infrared Spectra Analysis

The FTIR spectrum (Figure 2) exhibited characteristic bands of glucan polysaccharides. The broad intensity bands at 3300 cm^–1^ (LAS-N and LAS-S1) and 3464 cm^–1^ (LAS-S2) correspond to the O–H stretching vibration.^34^ The reduced intensity at 3464 cm^–1^ in LAS-S2 suggests a decrease in the number of hydroxyl groups after sulfation, consistent with the observation reported by Hao et al.^22^ of a similar decrease at 3197 cm^–1^ in polysaccharides from the agaric mushroom *Stropharia rugosoannulata*. In contrast, the low degree of sulfation in LAS-S1 did not significantly affect the absorption in this spectral region.

**Figure 2:**
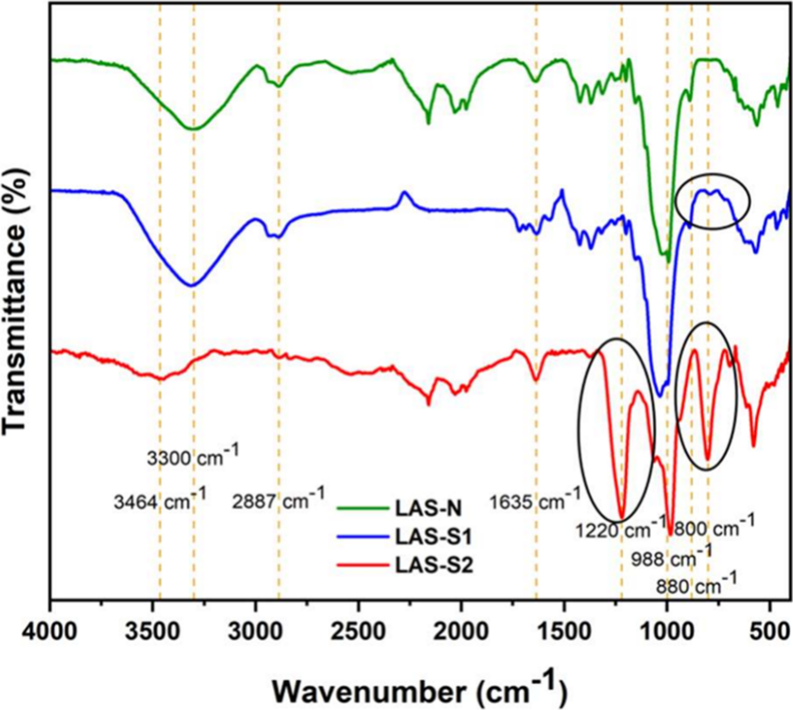
FTIR spectra of native LAS (LAS-N) and sulfated derivatives (LAS-S1 and LAS-S2).

The absorption band at 2887 cm^–1^, observed in all of the LAS compounds, corresponds to C–H stretching in sp^3^-hybridized carbon atoms, a characteristic of glucans. The low-intensity band at 1635 cm^–1^ is attributed to carbonyl stretching and/or water absorption.^35,36^ The region between 1400 and 1200 cm^–1^ reveals C–H and O–H bending vibrations, characteristics of carbohydrates, with minor intensity and wavenumber variations depending on the polysaccharide structure.^37^

The band at 1220 cm^–1^ indicates S=O stretching, while the band at 800 cm^–1^ corresponds to C–O–S bending,^22^ confirming sulfation, especially in the LAS-S2 (DS 0.51) sample. A weak band at 800 cm^–1^ in LAS-S1 reflects its lower degree of sulfation (DS = 0.11). The band at 998 cm^–1^ corresponds to glycosidic linkages, which may shift with changes in polysaccharide conformation,^38^ and the band at 880 cm^–1^ represents β-type glycosidic linkages, characteristic of β-glucan.^19^

### 3.2 Biological Activities

#### 3.2.1 Antioxidant Activity of Native and Sulfated Derivatives of LAS

Antioxidant activity is crucial for protecting cells from damage caused by reactive oxygen/nitrogen species (ROS/RNS).^39^ Excessive ROS accumulation or failures in cellular antioxidation result in oxidative stress, damaging proteins, lipids, and DNA, which accelerate cellular aging and contribute to chronic diseases, such as cancer and cardiovascular diseases.^40^ Methods employed to measure antioxidant activity have been reviewed extensively,^41^ and among them were the removal of hydroxyl radicals (·OH) and H_2_O_2_.

Lasiodiplodan [(1→6)-β-glucan] produced by *L. theobromae* MMPI demonstrated the ability to neutralize free radicals,^42^ preserve cellular integrity, and stimulate efficient immune responses.^43^ In this study, the antioxidant activities of LAS-S1 and LAS-S2 were compared to LAS-N in terms of the hydroxyl radical (·OH) and the pro-oxidant H_2_O_2_. Sulfation treatment did not significantly (*p* < 0.05) alter ·OH removal at higher concentrations (25 mg/mL), with values of 45, 42, and 44% observed for LAS-S1, LAS-S2, and LAS-N, respectively (Figure 3A) However, at 15 mg/mL, LAS-S1 exhibited approximately 10% higher antioxidant activity compared to LAS-N, highlighting the positive effect of moderate sulfation (DS 0.11). In contrast, LAS-S2, with a higher sulfonate group density (DS 0.51), showed lower antioxidant activity at 10 mg/mL, suggesting that excessive sulfonation treatment may impair antioxidant efficacy. Similarly, Chen et al.^32^ reported enhanced antioxidant properties in polysaccharides derived from the red alga *Pyropia yezoensis* sulfonated by CSA-Py, while Gunasekaran et al.^44^ also found comparable improvements in antioxidant activity with polysaccharides from *Pleurotus eous* sulfated by CSA-Py.

**Figure 3:**
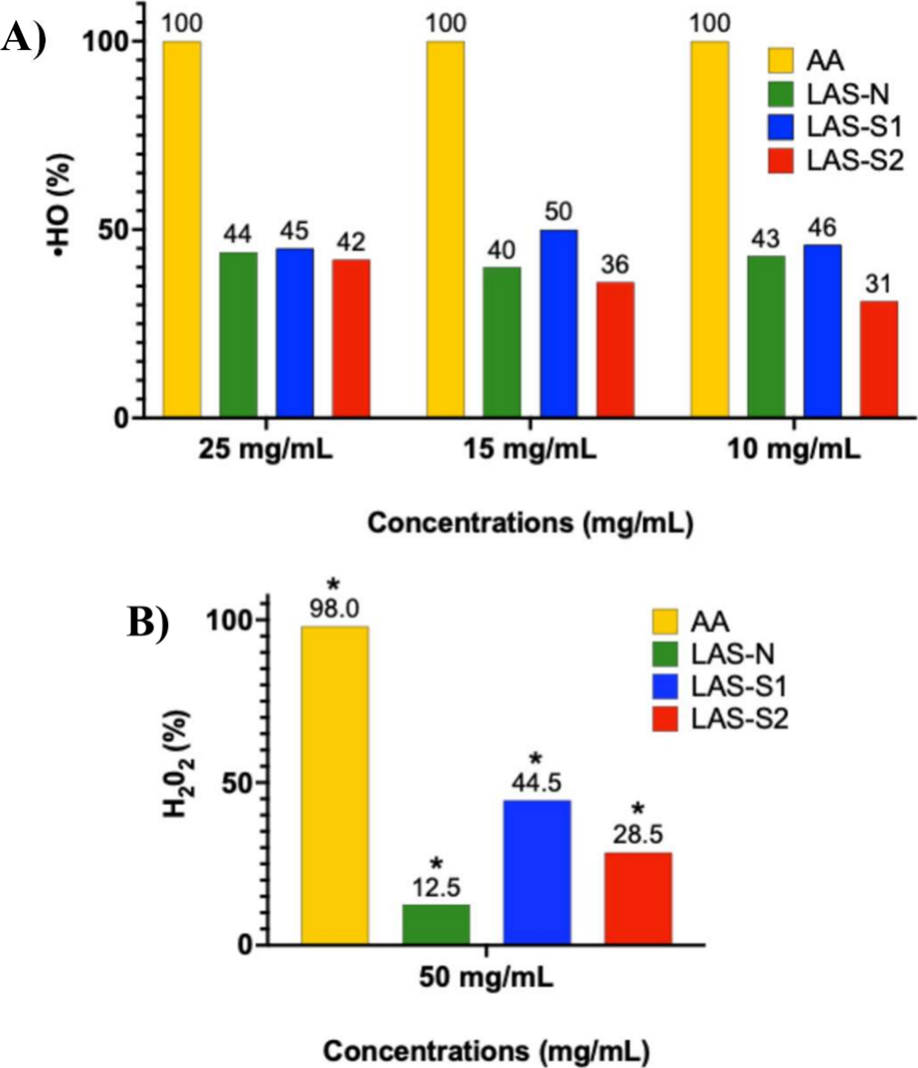
Removal potential of the hydroxyl radical (A) and hydrogen peroxide (B) of the samples LAS-N, LAS-S1, and LAS-S2 compared with the antioxidant standard ascorbic acid (AA). (*) indicates a *p-*value <0.05.

Sulfation treatment improved the H_2_O_2_ removal capacity compared to that of LAS-N (Figure 3B). LAS-S1 outperformed LAS-N at all tested concentrations, removing 44.5% of H_2_O_2_ at 50 mg/mL, compared to 12.5% for LAS-N and 28.5% for LAS-S2 (*p* < 0.05). The superior performance of LAS-S1 highlights the importance of intermediate levels of sulfonation in enhancing antioxidant activity, particularly at higher concentrations. The results suggest that sulfation could be an effective strategy to enhance ·OH and H_2_O_2_ scavenging ability, with a dose-dependent effect related to both the concentration of the sulfated compound and its degree of sulfation.

#### 3.2.2 Microbiostatic and Microbicidal Activities of Native and Sulfated Lasiodiplodan

The minimum inhibitory concentration (MIC) is the lowest concentration that can inhibit the growth of a bacterial or fungal species or strain. It is an essential parameter in determining sensitivity to a possible antimicrobial agent.^45^ In this study (Table 2), we observed that LAS-N demonstrated antimicrobial activity against *C. albicans*, *C. tropicalis*, and *E. coli*, with fungistatic and bacteriostatic effects up to 0.25 mg/mL. These findings are consistent with those obtained by Calegari et al.,^37^ who reported antimicrobial activity of LAS-N against *C. albicans* and *C. tropicalis*. The effects against these yeasts were more pronounced at higher concentrations tested, suggesting that LAS-N may exhibit variable efficacy across microbial species and experimental conditions.

**Table 2:** Antimicrobial Activity Exhibited by LAS-N and Its Sulfated Derivatives (LAS-S1 and LAS-S2) against Bacterial and Yeast Species^a^

	compounds		
	LAS-N	LAS-S1 (mg/mL)	LAS-S2
	degree of substitution		
microbial species and strain	0	0.11	0.51
*Candida albicans* 10231	# (0.25)	# (0.25)	# (0.25)
*C. tropicalis* 13803	# (0.06)	# (0.06)	+ (0.03)
*Escherichia coli* 25922	# (0.25)	# (0.25)	# (0.25)
*Listeria monocytogenes* 19111	-	-	# (0.25)
*S. enterica* Typhimurium 14008	-	-	+ (0.05)
*Staphylococcus aureus* 25923	-	-	-

a # Bacteriostatic (minimum bactericidal concentration, MBC)/fungistatic (minimum fungal concentration MFC) - inhibition of microbial growth. + Bactericidal (MBC)/fungicidal (MFC) - microbial cell death. - No inhibition observed.

LAS-S1 exhibited the same inhibitions as LAS-N, with fungistatic (*C. albicans* - MIC of 0.25 mg/mL, and *C. tropicalis* - MIC of 0.06 mg/mL) and bacteriostatic activity (*E. coli* - MIC of 0.25 mg/mL). These results indicated that the lower degree of sulfation of LAS did not improve antibacterial and antifungal activities. By contrast, LAS-S2 displayed a more pronounced antimicrobial profile. In addition, we observed fungicidal activity against *C. tropicalis* (MIC of 0.03 mg/mL), bacteriostatic activity against *L. monocytogenes* (MIC of 0.25 mg/mL), and bactericidal activity against *S. enterica* Typhimurium (MIC of 0.05 mg/mL). These findings demonstrate that the degree of sulfation in β-glucan directly influences its antimicrobial efficacy, particularly against Gram-negative bacteria, such as *S. enterica*, and the yeast *C. tropicalis*, which are more sensitive to LAS-S2. These effects could be attributed to the increased negative charge of the sulfate groups, which facilitates their interaction with the bacterial cell wall. The resistance observed in *S. aureus* is consistent with the literature, which has reported this pathogen to be resistant to various antimicrobial agents.^46^ The lack of effect against *S. aureus* can be attributed to the bacterium’s natural resistance to sulfated agents, consistent with observations reported by Calegari et al.^19^

Sulfation of β-glucan, like that of chitosan, is widely recognized for enhancing its antimicrobial activity, especially against Gram-negative bacteria, by overcoming the protection provided by the outer membrane.^47^ The outer membrane structure in Gram-negative bacteria hinders the action of antimicrobial compounds, as evidenced by LAS-N, which showed limited interaction with these bacterial cells. However, the results of this study suggest that sulfation of lasiodiplodan (LAS-S2) overcame this barrier, promoting more effective interaction with microbial cells. These findings (Table 2) support the hypothesis that sulfation may be a key factor in enhancing antimicrobial efficacy against Gram-negative bacteria and fungi.

#### 3.2.3 Cytotoxicity and Anti-RSV Activity

Concerns regarding toxicity, safety, and the costs of treatment have increasingly restricted the use of antiviral agents, such as ribavirin [64], paving the way for the development of vaccines and monoclonal antibodies to treat human RSV infections. Continuing studies on the use of natural products, such as bioactive polysaccharides, particularly sulfated ones, have demonstrated significant therapeutic potential against major human pathogenic viruses due to their structural characteristics that facilitate viral interactions.^48^ With this in mind, this study reports, for the first time, the antiviral activity of sulfated derivatives LAS-S1 (synthesized using Protocol 1: CSA-DMF) and LAS-S2 (Protocol 2: CSA-Py) against RSV, a significant causative agent of human respiratory tract infections.

First, we tested the native and sulfated LAS compounds for cytotoxicity in the HEp-2 cell line. All the compounds exhibited no cytotoxicity at the highest tested concentration (1000 μg/mL), with viable percentages ranging from 84.46 to 97.22%, 78.56 to 100%, and 71.59 to 87.77%, respectively, for LAS-N, LAS-S1, and LAS-2 (Figure 4).

**Figure 4:**
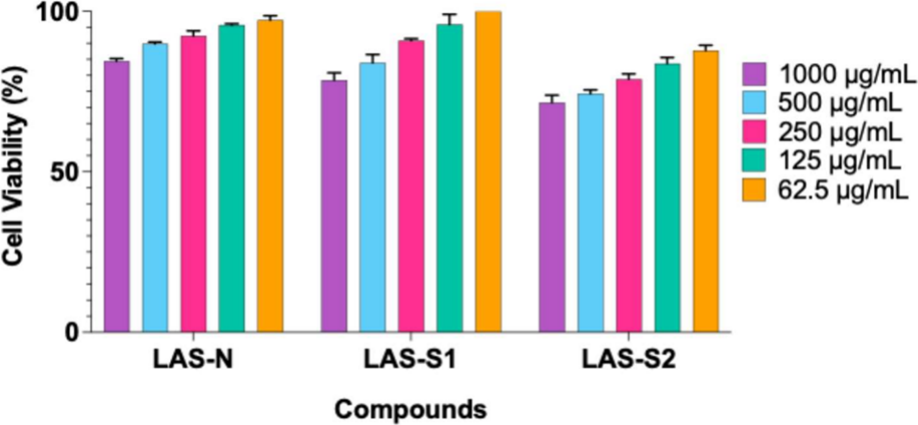
Cell viability percentage (%) of HEp-2 cells in response to different concentrations (62.5–1000 μg/mL) of native lasiodiplodan (LAS-N) and its sulfonated derivatives (LAS-S1 and LAS-S2), as assayed by the MTT procedure. Standard deviations are represented by error bars.

The CC_50_ values for HEp-2 cells were greater than 1000 μg/mL for both native and sulfated lasiodiplodans (Table 3). In a related study, Wouk et al.^20^ also reported a CC50 greater than 1000 μg/mL for LAS-N in Vero cells (African green monkey kidney cells). In that study, sulfation was performed differently (chlorosulfonic acid in pyridine at different ratios) to produce sulfated LAS compounds (DS of 0.18 to 1.61) that presented CC_50_ ranging from 125 μg/mL (DS 0.18) to 537.5 μg/mL (DS 1.61) and differed from the results obtained in the present study for LAS-S1 (DS 0.11) and LAS-S2 (DS 0.51). The protective effect of the sulfated LAS compounds was greater than that of ribavirin (an antiviral commonly used to treat RSV infection), which had a much lower CC50 of 230.6 μg/mL.

**Table 3:** Cytotoxicity Exhibited by Native Lasiodiplodan (LAS-N) and Its Sulfated Derivatives (LAS-S1 and LAS-S2) against the HEp-2 Cell Line and Antiviral Activity against Respiratory Syncytial Virus (RSV)^a^

compounds	DS^b^	CC_50_ ^c^	IC_50_ ^d^	SI^e^
LAS-N	0.00	>1000	>200	>5
LAS-S1	0.11	>1000	34.5 ± 4.56	>28.98
LAS-S2	0.51	>1000	1.38 ± 5.88	>724.63
ribavirin	0.00	230.6	1.21	189.32

a Ribavirin was used as a positive control.

b DS: degree of substitution with sulfate groups.

c CC_50_, 50% inhibitory concentration of LAS compounds toward the viability of HEp-2 cells (μg/mL).

d IC_50_, 50% inhibitory concentration of viral infectivity (μg/mL).

e SI, selectivity index (CC_50_/IC_50_). The results are expressed as mean ± standard deviation.

When the sulfated compounds LAS-S1 and LAS-S2 were evaluated for anti-RSV activity, LAS-N inhibited only 27% of RSV replication in HEp-2 cells at the highest concentration tested (200 μg/mL), indicating a low antiviral effect in its native form (Figure 5). In contrast, sulfated derivatives inhibited RSV infection by more than 50%. The best anti-RSV effect was demonstrated by LAS-S2, inhibiting RSV by 93, 90, 84, 78, and 70% at concentrations of 200, 100, 50, 25, and 12.5 μg/mL, respectively. At the same concentrations, LAS-S1 exhibited moderate antiviral effects (66, 61.78, 53.89, 47, and 38%).

**Figure 5:**
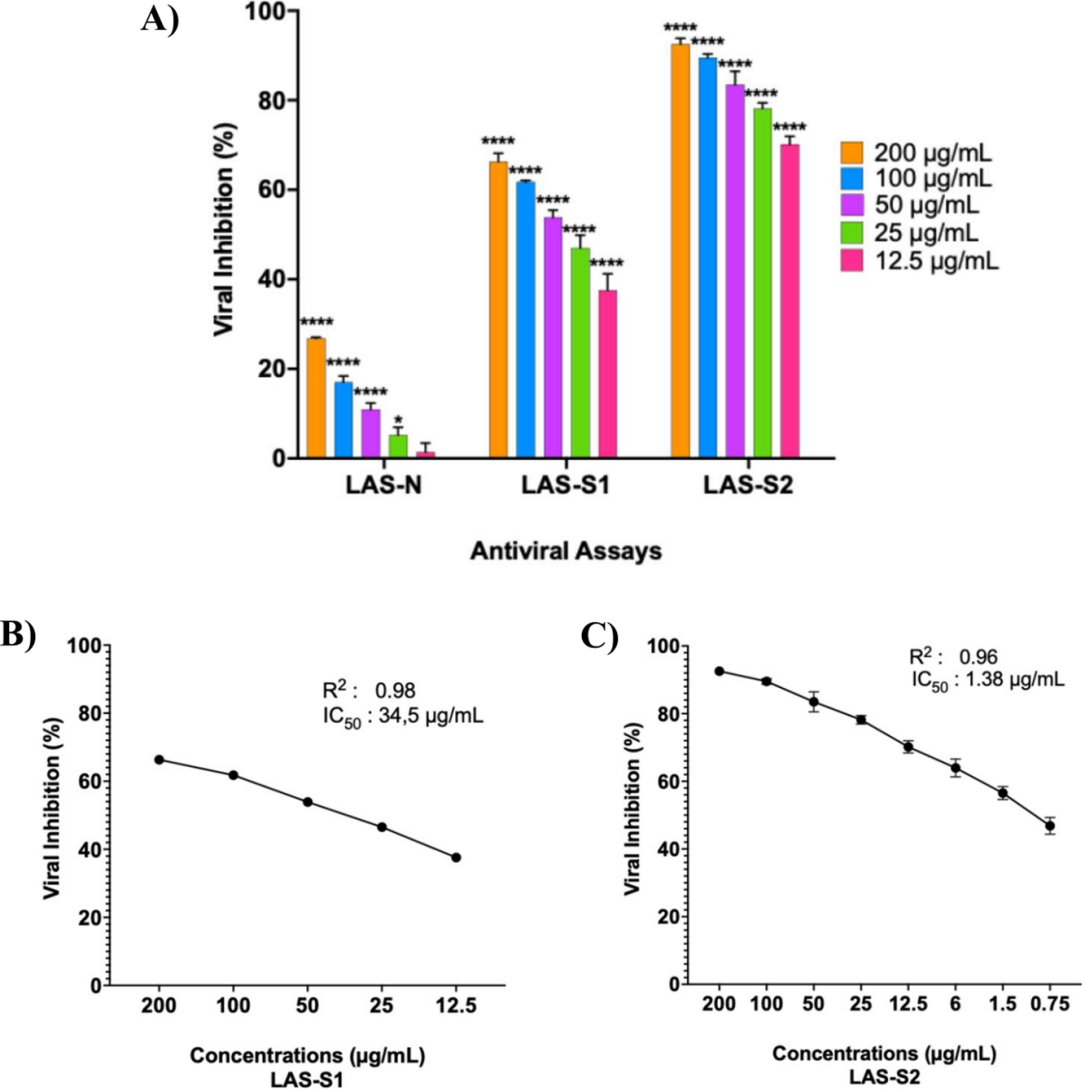
(A) Percentage of respiratory syncytial virus inhibition (%VI) in HEp-2 cells exposed to different concentrations (μg/mL) of LAS-N, LAS-S1, and LAS-S2. Inhibition percentages were calculated relative to controls (uninfected and untreated or infected and untreated). Dose–response curves (0 h) for LAS-S1 (B) and (C) LAS-S2, showing IC_50_ values and *R* ^2^ values generated by linear regression. The concentrations in each treatment were compared by two-way analysis of variance (ANOVA), followed by Dunnett’s test. Standard deviations are represented by error bars. The presence of an asterisk (****) indicates a *p-*value <0.0001.

Among the tested sulfated compounds, LAS-S2 showed greater efficacy against RSV (IC_50_ of 1.38 μg/mL and SI > 724.63), whereas LAS-S1, by contrast, exhibited moderate antiviral activity (IC_5_0 of 34.5 μg/mL; SI > 28.98), while LAS-N resulted in IC_5_0 > 200 μg/mL and SI > 5) (Table 3). This finding indicates that LAS-S2 exerts a highly potent inhibitory effect compared with LAS-N and LAS-S1, with an efficacy similar to that of ribavirin (IC50, 1.218 μg/mL). LAS-S2 (DS 0.51) exhibited the best antiviral performance, suggesting that differences in sulfation degree influence its efficacy. Our results corroborate those of previous studies by Wouk et al.^20^ that identified an LAS-S of DS 1.61 as the most effective compound against Herpes simplex virus HSV-1: IC_50_/SI values of 6.1 μg/mL/88.1 for acyclovir-sensitive (KOS), and 2.8 μg/mL/189.2 for acyclovir-resistant (AR) viral strains. Pires et al., 2023 independently evaluated the anti-RSV activity of carboxymethylated derivatives of LAS from a different strain of *L. theobromae* (MMBJ) and found viral inhibition (IC_5_0 of 0.7 μg/mL), but with a lower SI value (CC_50_ of 1.4 μg/mL and SI: 2).^49^

The Selectivity Index (SI) was calculated from cytotoxicity (CC_50_) and viral inhibition (IC_50_) values. The SI of LAS-S2 for RSV as the primary target is exceptionally high, with a value exceeding 724.63; approximately four times higher than the SI of ribavirin (189.32). By contrast, the SI of LAS-S1 for RSV was considerably higher (>28.98) than that of LAS-N.

Compounds with SI ≥ 10 are considered active, and their biological efficacy does not correlate with in vitro cytotoxicity at SI > 10. However, it is crucial to note that even compounds with SI < 10 may demonstrate efficacy in animal models. In vitro SI values play an essential role in identifying active compounds for the future development of drugs.^50,51^

LAS-S2 combines robust antiviral efficacy with low toxicity and selectivity, positioning it as a promising candidate for clinical investigation. Therefore, optimizing sulfation processes and obtaining a degree of sulfation, such as by the CSA-Py protocol (2) reported here, could present a promising strategy for the development of more effective antiviral compounds.

#### 3.2.4 Mode of Action of LAS-S2 against RSV Replication

The initial phase of viral entry into host cells involves adsorption, followed by structural alterations in both the virus and the host cell. These changes are essential for initiating viral internalization, a process characterized by penetration and uncoating that leads to the release of viral genetic material. The replication of new viral particles relies on host cellular enzymes and transcription factors, followed by the assembly of nucleocapsids, the acquisition of viral envelopes, and the release of new virions.^52^

The inhibitory effect of LAS-S2 was dose-dependent across all treatment protocols (Figure 6). In the pretreatment procedure, when LAS-S2 was added over a longer period (−3 h), the sulfated compound showed greater inhibition (93, 89, 79, and 48% at 200 μg/mL, 100, 50, and 25 μg/mL, respectively) compared to −1 h (ranging from 77% at 200 μg/mL to 52% at 25 μg/mL) (*p* < 0.001). These results reinforce LAS-S2’s prophylactic effect, demonstrating its ability to reduce viral infectivity before RSV enters cells. When LAS-S2 was added at times +1 and +3 h (post-treatment), inhibition ranged from 66 to 57% and 90 to 54%, respectively, between concentrations of 200 and 25 μg/mL, also demonstrating its therapeutic potential (*p* < 0.001). The inhibitory effect in the extracellular environment was performed by adding LAS-S2 in contact with RSV for 1 h (virucidal effect), obtaining high percentages of inhibition (83, 72, 64, and 62%) at all tested concentrations (200, 100, 50, and 25 μg/mL, respectively) (*p* < 0.001) (Figure 6). This result suggests that LAS-S2 can directly inactivate RSV. To complement the possible initial stages of infection in which LAS-S2 could act, adsorption and penetration inhibition assays were performed. LAS-S2 showed strong inhibition at both stages of virus entry into the cell, with inhibition ranging from 90 to 81% in adsorption and from 69 to 58% in penetration at concentrations of 200 to 25 μg/mL (*p* < 0.001).

**Figure 6:**
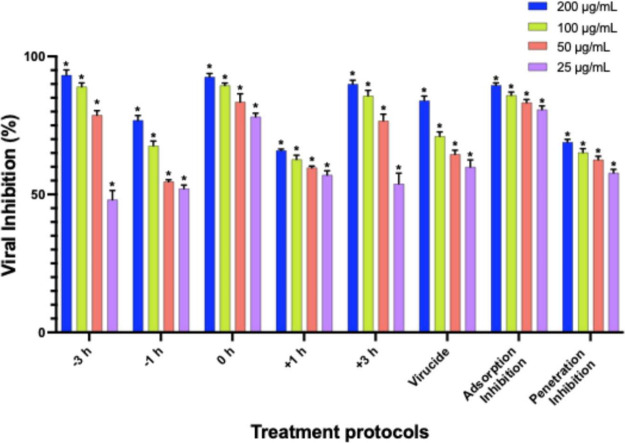
Evaluation of the anti-RSV mechanism of action of LAS-S2. The sulfated compound (200–25 μg/mL) was assessed at different stages of RSV replication: time of addition (HEp-2 cells were exposed to LAS-S2 for −1 and −3 h (pretreatment) before, 0 h (simultaneous treatment), or +1 and +3 h (post-treatment) after viral inoculation); virucidal assay (LAS-S2 was incubated directly with the virus for 1 h); adsorption (HEp-2 cells were preincubated with LAS-S2 added simultaneously with RSV; and penetration (after preincubation with LAS-S2, the HEp-2 cells were inoculated with RSV). The percentage of viral inhibition was calculated by comparing the controls (CC = uninfected and untreated; VC = infected and untreated). The concentrations in each treatment were compared by two-way analysis of variance (ANOVA), followed by Dunnett’s test. The standard deviation is represented by error bars. The presence of an asterisk (*) indicates a *p*-value <0.001, demonstrating statistical significance when the test means are compared to the viral control (VC) mean.

Structural modification of polysaccharides by the addition of functional groups, such as sulfates, can significantly enhance their water solubility and alter their antiviral efficacy. Sulfated polysaccharides can exhibit various mechanisms of action, including the prevention of infection and antiviral activity within cells.^53^ The primary mechanism involves interactions, such as electrostatic, hydrophobic, and polar forces, with either the virus or the target cell. These interactions are influenced by factors such as the DS, molecular weight, and specific structural features, including the presence and arrangement of sulfate groups.^54^ Such interactions can neutralize the positive charges on viral proteins, leading to a virucidal effect that hinders the virus’s ability to recognize and bind to cellular receptors. Additionally, sulfated polysaccharides can bind to the host cellular receptor directly, thereby inhibiting viral adsorption. Compounds with these properties are crucial for managing the transmission and spread of viral infections.

Sulfated polysaccharides may also inhibit processes occurring after adsorption, thereby disrupting the structural changes necessary for the virus to bind to allosteric sites on its host’s receptors. Such interference can prevent penetration, uncoating, or the final release of the virus from the host cell.^55^ Furthermore, these compounds may indirectly inhibit viral transcription and replication by interacting with cellular proteins or receptors, thereby modulating intracellular processes. This modulation may interfere with enzymes essential for viral replication or stimulate the production of proinflammatory cytokines, thereby inducing an antiviral state within the host cell. Overall, compounds exhibiting these activities play a crucial role in controlling viral replication within host cells, thereby facilitating effective infection management.

The mechanisms described above are particularly relevant for LAS-S2, suggesting its antiviral effects during the early and intermediate stages of infection and possibly providing a new alternative to the reference drug (ribavirin). Although ribavirin remains a reference antiviral drug and is often considered a gold standard for RSV treatment, its clinical use is limited by well-documented adverse effects including dose-dependent toxicity and hematological complications. These safety concerns have restricted its widespread application and underscore the need for alternative antiviral agents with improved safety profiles. In this context, the sulfated derivative LAS-S2 demonstrated potent anti-RSV activity in vitro, with no detectable cytotoxicity at the highest tested concentration, resulting in a markedly higher selectivity index than ribavirin. These findings suggest that LAS-S2 may offer a favorable therapeutic window and highlight its potential translational relevance as a safer antiviral candidate, warranting further preclinical investigation.

## 4 Conclusions

This study investigated the effects of chemical sulfation on the structure and biological properties of (1→6)-β-d-glucan (lasiodiplodan), a fungal exopolysaccharide. Sulfation was performed using two distinct methods, CSA-DMF and CSA-Py, resulting in different degrees of substitution (DS): 0.11 for LAS-S1 and 0.51 for LAS-S2. Chemical characterization revealed significant structural modifications in the native EPS (LAS-N), which were characterized by the appearance of sulfation-specific bands in the FTIR spectra. Regarding its biological properties, sulfation of native lasiodiplodan increased its solubility, and the sulfated derivatives enhanced antioxidant and antimicrobial activities. LAS-S1, with a lower DS, demonstrated a greater ability to eliminate ·OH and H_2_O_2_ compared to LAS-N and LAS-S2. By contrast, LAS-S2 stood out for its antimicrobial activity, showing potent fungicidal activity against *C. tropicalis* (MIC = 0.03 mg/mL), bacteriostatic activity against *L. monocytogenes* (MIC = 0.25 mg/mL), and bactericidal activity against *S. enterica* Typhimurium (MIC = 0.05 mg/mL). The best effect of LAS-S2 was also demonstrated in its anti-RSV activity, which acts at different stages of viral replication: directly on the viral particle (virucidal effect), at early stages of viral replication (pretreatment, adsorption, and penetration), and postinfection. Compared to ribavirin, LAS-S2 showed higher efficacy and is regarded as safe (IC_50_, 1.38 μg/mL; Selectivity Index, SI > 724.63). These results suggest that LAS-S2, with lower antioxidant activity but excellent antimicrobial and antiviral activity, is a promising therapeutic agent against RSV. To maximize its therapeutic potential, future studies should examine how varying degrees of sulfation affect molecular interactions with specific viral targets and consider crucial factors, such as the bioavailability and cytotoxicity of the tested compounds in host cells.

## Data Availability

The data supporting the findings of the present study are available upon request.
